# Evolutionary tracking is determined by differential selection on demographic rates and density dependence

**DOI:** 10.1002/ece3.6311

**Published:** 2020-06-01

**Authors:** Anna Christina Vinton, David Alan Vasseur

**Affiliations:** ^1^ Department of Ecology and Evolutionary Biology Yale University New Haven Connecticut

**Keywords:** climate change, demography, eco‐evolution, evolutionary rescue, evolutionary tracking, extinction, life‐history traits

## Abstract

Recent ecological forecasts predict that ~25% of species worldwide will go extinct by 2050. However, these estimates are primarily based on environmental changes alone and fail to incorporate important biological mechanisms such as genetic adaptation via evolution. Thus, environmental change can affect population dynamics in ways that classical frameworks can neither describe nor predict. Furthermore, often due to a lack of data, forecasting models commonly describe changes in population demography by summarizing changes in fecundity and survival concurrently with the intrinsic growth rate (*r*). This has been shown to be an oversimplification as the environment may impose selective pressure on specific demographic rates (birth and death) rather than directly on *r* (the difference between the birth and death rates). This differential pressure may alter population response to density, in each demographic rate, further diluting the information combined to produce *r*. Thus, when we consider the potential for persistence via adaptive evolution, populations with the same *r* can have different abilities to persist amidst environmental change. Therefore, we cannot adequately forecast population response to climate change without accounting for demography and selection on density dependence. Using a continuous‐time Markov chain model to describe the stochastic dynamics of the logistic model of population growth and allow for trait evolution via mutations arising during birth events, we find persistence via evolutionary tracking more likely when environmental change alters birth rather than the death rate. Furthermore, species that evolve responses to changes in the strength of density dependence due to environmental change are less vulnerable to extinction than species that undergo selection independent of population density. By incorporating these key demographic considerations into our predictive models, we can better understand how species will respond to climate change.

## Graphical abstract

Evolutionary rescue, or the ability of a population to avoid extinction via genetic adaptation, is already known to greatly increase the chances of persistence and it is clear that the initial population size, genetic variation, rate of environmental change, and dispersal are important predictors of its success. There is a current lack a general understanding of how these predictors are linked to the demographic rates and traits that are under selection during evolutionary rescue. We employ a common model of population dynamics to investigate how different assumptions about the relationship between life‐history traits and the environment change the propensity for evolutionary tracking, and ultimately evolutionary rescue to occur.

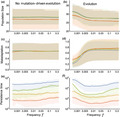

## 1 INTRODUCTION

Environmental change can lead to extinction when population growth rates decline to negative values or when carrying capacities are sufficiently small to generate stochastic extinctions. The ability of a population to persist following environmental change requires a shift back to positive growth rates via ecological or evolutionary mechanisms. The need to understand the mechanisms underlying population rebound has spurred studies about how demographic rescue (via immigration) and genetic rescue (via an increase in genetic variation) (Brown & Kodric‐Brown, 1977; Hufbauer et al., 2015; Whiteley, Fitzpatrick, Funk, & Tallmon, 2015) allow for population rebound after an environmentally driven decline. Evolutionary rescue (population rebound due to an increase in density of an adaptive genotype), in particular, provides a lens to investigate extinction which incorporates both the demographic and evolutionary components of population rebound postenvironmental change (Gonzalez, Ronce, Ferriere, & Hochberg, 2013).

The search for what makes evolutionary rescue possible has led to an increasing effort to find experimental, empirical, and theoretical evidence of this phenomenon (Bell & Gonzalez, 2009, 2011; Gomulkiewicz & Holt, 1995; Gonzalez et al., 2013; Johannesson, Smolarz, Grahn, & André, 2011; Lindsey, Gallie, Taylor, & Kerr, 2013; Lynch, Wilfried, & Wood, 1991; Martin, Aguilée, Ramsayer, Kaltz, & Ronce, 2013; Martin‐Clemente, Melero‐Jiménez, Bañares‐España, Flores‐Moya, & García‐Sánchez, 2019; Mills et al., 2018; Orr & Unckless, 2008; Ramsayer, Kaltz, & Hochberg, 2013; Zhang & Buckling, 2011). (Bell & Gonzalez, 2009) identified four factors which alter the propensity for evolutionary rescue: initial population size, genetic variability due to standing genetic variation and mutations, genetic variability due to dispersal, and the extent and severity of environmental change. These four factors have been confirmed in numerous empirical and experimental studies (Anciaux, Chevin, Ronce, & Martin, 2018; Lindsey et al., 2013; Martin et al., 2013; Mills et al., 2018; Ramsayer et al., 2013). Although these results have advanced our understanding of the necessary conditions for evolutionary rescue, we still lack a clear understanding of the role of the underlying demographic rates in mediating the outcome (Anciaux et al., 2018), since these demographic rates may have complex relationships with the four factors described above and with their response to the environment.

### 1.1 Selection can vary birth and death rates and determine adaptive capability

Environmental change can stress populations and reduce population growth rate by decreasing the birth rate, increasing the death rate, or some combination of the two (Aanes, Sæther, & Øritsland, 2000; Barfield, Holt, & Gomulkiewicz, 2011; Brewer & Peltzer, 2009; Clutton‐Brock & Coulson, 2002; Crump, Hopkinson, Sogin, & Hobbie, 2004; Dempster, 1983; Mccredie, Malcolm, Fred, & Charles, 1983; Sibly, Barker, Denham, Hone, & Pagel, 2005; Sibly, Williams, & Jones, 2000). To generalize across taxa, previous studies investigating evolutionary rescue commonly model demographic rates using deterministic models that do not differentiate how the environment acts on the birth and death rates, but rather use a fixed parameter, the intrinsic rate of population increase, *r* (the difference between the birth rate and the death rate at low density) (Lynch et al., 1991). Consequently, information about changes in a particular demographic rate can be lost if *r* is the focus of a study.

Populations with the same *r*, but different underlying demographic rates, can respond quite differently to environmental selection (Holt, 1990). Take the case of two populations, where one has a high birth and death rate, while another has a low birth and death rate. If the difference between the two rates is equal, both populations will have the same *r*. But, all else held equal, the population with the higher birth and death rate will have a faster rate of population turnover and will evolve in response to selection more quickly than the population with the low birth and death rate. Thus, the potential for successful evolutionary tracking of small populations depends explicitly on birth and death rates, not *r*, which abstracts away from these rates and obscures the actual speed of adaptation by ignoring the rate of population turnover.

Furthermore, both deterministic (predictable, e.g., adaptive evolution) and stochastic (unpredictable, e.g., genetic drift) processes determine evolutionary rescue (Lande, Engen, & Saether, 2003). Although adaptive evolution favors individual traits that are best suited to their environment, stochastic processes can lead to dissimilarities from predictions produced by purely deterministic frameworks (Start, Weis, & Gilbert, 2019; Vellend, 2016). Moreover, demographic stochasticity (the randomness resulting from individual variation in birth and death, as well as variation in the timing of birth and death) influences the ability of evolution to favor adaptation to a changing environment. At large population sizes, these individual differences average out, yet they remain important at small population sizes (Lande et al., 2003; May, 1973). For this reason, modeling this demographic stochasticity explicitly is especially relevant when considering the ability of populations to rebound from small size. The importance of modeling birth and death rates explicitly has thus been emphasized widely, leading to a wide use of theoretical modeling techniques that incorporate stochasticity into birth and death rates (DeLong & Gibert, 2016; Melbourne & Hastings, 2008; Nåsell, 2001; Ovaskainen & Meerson, 2010). Similarly, population demographers are increasingly able to collect data on how different vital rates change over time, making it especially timely to incorporate these nuances into our predictive models (Coulson et al., 2001; Ouyang et al., 2014; Sibly et al., 2000).

### 1.2 Selection can alter how density dependence acts in populations, determining probability of rebound

Density dependence can also affect birth and death rates and has been shown to influence the dynamics of many species (Coulson et al., 2001; Ouyang et al., 2014; Reed & Slade, 2008; Sibly et al., 2000). Environmental change may or may not alter the strength of density dependence; this varies across taxa and type of change (Coulson et al., 2001; Owen‐smith, 1990; Sibly et al., 2000). For example, environmental change, such as drought, may decrease the availability or accessibility of water‐limited resources (Owen‐smith, 1990), intensifying density dependence as individuals compete for said resources. Theoretical studies predict that compensatory density dependence, a decrease in growth rate at high densities and increase at low densities, would allow for a larger population size following environmental change (Chevin & Lande, 2010; Ferguson & Ponciano, 2015; Holt, 1990; Lande et al., 2003), further facilitating adaptation to new environments. Furthermore, the rate of rebound from small population size has been shown to be proportional to the extent of density dependence (Chevin & Lande, 2010; Lande et al., 2003). Thus, establishing the interaction between density dependence and environmental change in different demographic rates is of the utmost importance as the population size following an environmental perturbation determines the probability of extinction. Density dependence, although often difficult to quantify in part due to the time delay in its appearance (Lande et al., 2003; May, 1973), has also been increasingly taken into account by demographers. How density dependence evolves due to selection imposed by environmental change is for the most part only recently being brought into eco‐evolutionary models and is absent from models of evolutionary rescue (Martin et al., 2013).

Studies of evolutionary rescue focusing on *r* need to be expanded because (a) the underlying demographic parameters and (b) the interaction between environmental change and density dependence may strongly affect evolution. We investigate how evolutionary tracking, and furthermore evolutionary rescue, depends on the way environmental change affects population demographic rates. Here, we incorporate environmental conditions and their effects on density dependence into per capita rates of birth and death, to elucidate their effect on population dynamics and persistence in a stochastic model. We show results using both a fluctuating environment and a nonfluctuating environment with a single environmental shift to emphasize the roles of ecological and evolutionary tracking and to extend our results to evolutionary rescue. We find that populations where the environment affects their death rate as opposed to their birth rate are more vulnerable to extinction. Furthermore, when environmental change intensifies density dependence, populations are better able to rebound from small population sizes and evolutionarily track their changing environment.

## 2 METHODS

### 2.1 Model formulation

We construct a continuous‐time individual‐based logistic growth model and then consider four ways that environmental change might alter population demographic rates. In all cases, as these are logistic growth models, either the birth or death rate is density dependent. In Cases 1a and 1b, the environment alters the birth rate in a density‐independent and density‐dependent manner, respectively. In Cases 2a and 2b, the environment alters the death rate similarly, in a density‐independent and density‐dependent way. We pay particular attention to ensuring that the four cases converge on the same outcome when the environment is static, to best isolate the effects of life history and selection on evolutionary rescue.

### 2.2 Logistic growth

All of our model cases are rooted in the logistic growth equation where g(N) is a function describing the density dependence of the per capita growth rate and *N* represents the population density:

(1) $$\frac{\mathrm{dN}}{\mathrm{dt}}=g\left(N\right)N$$

g(N) is equal to the difference between the per capita birth rate *B* and the per capita death rate *D*, and we rewrite Equation (1) as follows:

(2) $$\frac{\mathrm{dN}}{\mathrm{Ndt}}=b_{0}-b_{I}-b_{D}N-\left(d_{0}+d_{I}+d_{D}N\right),$$

where *b_0_* and *d_0_* represent background rates of birth and death, *b_I_* and *d_I_* are density‐independent modifications to the birth and death rates, and *b_D_* and *d_D_* are density‐dependent modifications. Consistent with most derivations of logistic growth (Nåsell, 1996, 2001), and without loss of generality, we assume that density‐independent and dependent factors tend to reduce per capita birth rates and increase death rates. We fix the values of *b_0_* and *d_0_*, focusing on the density‐independent and density‐dependent modification terms as different modes of entry for environmental effects.

### 2.3 Environmental effect

We model the effect of the environment on the density‐independent and density‐dependent modification terms by calculating the mismatch between the environment and the trait of an individual. For simplicity and tractability, we first model the environment (or optimal trait value) $\mu_{\mathrm{opt}}$ as a simple sinusoidal function of time (see discussion for our reasons for this choice),

(3) $$\mu_{\mathrm{opt}}\left(t,f\right)=\cos\left(2\pi ft\right)$$

where *f* is frequency and *t* is time. The effect of the environment on an individual with trait value *μ* is then given by

(4) $$\varepsilon_{\mu }=\left|\mu -\mu_{\mathrm{opt}}\left(t,f\right)\right|$$

where a large $\varepsilon_{\mu }$ represents a maladapted individual, and a small $\varepsilon_{\mu }$ represents a well‐adapted individual. We systematically incorporate the environmental effect $\varepsilon_{\mu }$ into the density‐independent (*b_I_* and *d_I_*) and density‐dependent (*b_D_* and *d_D_*) components of the birth and death rates. However, to facilitate comparison among the model cases, we scale our equations so that for any value of $\varepsilon_{\mu }$ , the equilibrium population size (assuming no temporal environmental change) is the same across all of the model cases. This allows us to make an exact comparison of the impact of temporal environmental change on population dynamics, mediated by ecology and evolution. We accomplish this by assuming that a carrying capacity exists; this requires therefore that either the birth or death rate incorporates a nonzero density‐dependent effect ( $b_{D}\;\text{or}\;d_{D}\neq 0$ ). We define the carrying capacity for a population in which all individuals are perfectly adapted to the environment, equal to K_A_ (with K_A_ = 35). We introduce a second carrying capacity, K_B_ = 18 for a population that is two trait units from the optimum ( $\varepsilon_{\mu }$ = 2). This sets a quasi‐lower bound for the carrying capacity, since the amplitude of variation in the trait optima (Equation 4) is equal to one. Demographic and evolutionary stochasticity may however lead to instances where this is exceeded, but this does not compromise the integrity of the model.

We then independently solve the parameters *b_I_*, *b_D_*, *d_I_*, and *d_D_* given the conditions set above for the population carrying capacity and assuming that only one of *b_I_*, *b_D_*, *d_I_*, or *d_D_* will incorporate an environmental effect in each instance of the model. When the environment enters via a density‐independent effect on the birth rate (Case 1a) we find

(5) $$\left\{b_{I},b_{D}\right\}\;=\left\{\frac{K_{B}-K_{A}}{2K_{A}}\varepsilon_{\mu },\;\frac{b_{0}-d_{0}}{K_{A}}\right\}\;\text{and}\;\left\{d_{I},d_{D}\right\}=\left\{0,0\right\}$$

When the environment enters via a density‐independent effect on the death rate (Case 2a), the solutions for the birth and death parameters in equation 5 are switched. When the environment enters via a density‐dependent effect on births (Case 1b), we find

(6) $$\left\{b_{I},b_{D}\right\}=\left\{0,\;\left(\frac{b_{0}-d_{0}}{K_{A}}\right){\left(1+\frac{K_{B}-K_{A}}{2}\varepsilon_{\mu }\right)}^{-1}\right\}\;\text{and}\;\left\{d_{I},d_{D}\right\}=\left\{0,0\right\}$$

Similar to above, the model describing a density‐dependent effect on deaths (Case 2b) is found by switching solutions of the birth and death parameters in equation 6. Table 1 provides a breakdown of the model cases.

**Table 1:** Functional forms for the density‐independent (*b_I_* and *d_I_*) and density‐dependent (*b_D_* and *d_D_*) modifications to birth and death rates for Case 1a‐2b. The birth and death rates for each case are depicted in Figure 1 across a range of environmental conditions

Environment enters via	Density Independent		Density Dependent	
Birth	Case 1a	$b_{I}=\frac{K_{B}-K_{A}}{2K_{A}}\varepsilon_{\mu }$ $b_{D}\;=\frac{b_{0}-d_{0}}{K_{A}}\;$ $d_{I},d_{D}=0$	Case 1b	$b_{D}=\left(\frac{b_{0}-d_{0}}{K_{A}}\right){\left(1+\frac{K_{B}-K_{A}}{2}\varepsilon_{\mu }\right)}^{-1}$ $b_{I},d_{I},d_{D}=0$
Death	Case 2a	$d_{I}=\frac{K_{B}-K_{A}}{2K_{A}}\varepsilon_{\mu }$ $d_{D}\;=\frac{b_{0}-d_{0}}{K_{A}}\;$ $b_{I},b_{D}=0$	Case 2b	$d_{D}=\left(\frac{b_{0}-d_{0}}{K_{A}}\right){\left(1+\frac{K_{B}-K_{A}}{2}\varepsilon_{\mu }\right)}^{-1}$ $b_{I},b_{D},d_{I}=0$

**Figure 1:**
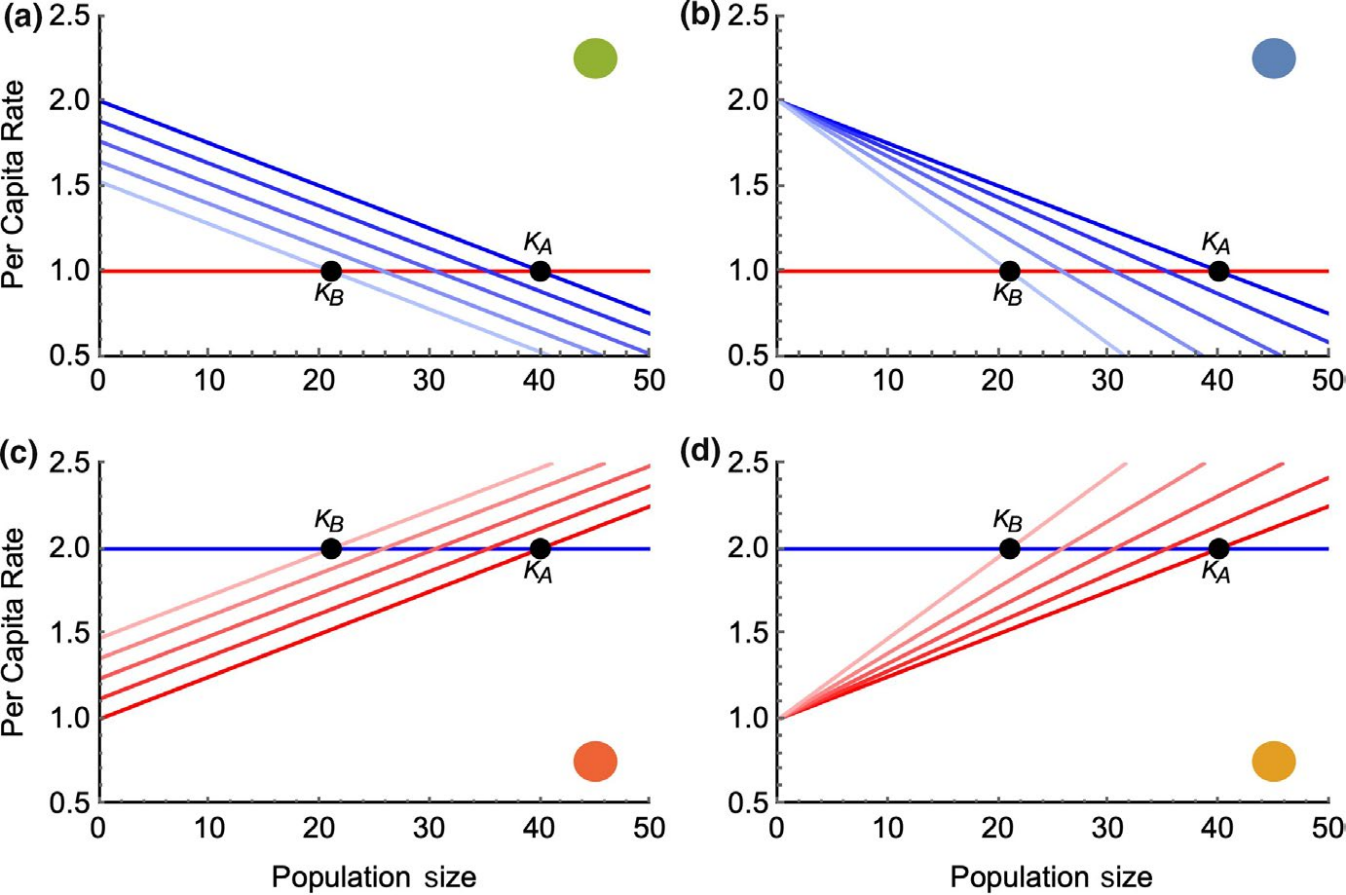
All four cases yield logistic population growth, but depend on different relationships between per capita demographic rates of birth (blue) and death (red) (see equations 5 and 6, and Table 1). In the upper panels (Cases 1a, 1b), death rate is constant, birth rate is density‐dependent, and the environment either directly increases or decreases birth rate (1a) or changes the strength of the relationship between density and birth rate (i.e., density dependence) (1b). Each blue line depicts the rate of birth for a particular state of adaptation to the environment, ranging from perfectly adapted $\varepsilon_{\mu }$ = 0, as dark blue (top line), to strongly maladapted $\varepsilon_{\mu }$ = 2, as light blue (bottom line). The lower panels show the same relationships for Cases 2a and 2b. Colored disks show how the 4 scenarios match to Figures 3 and 4

### 2.4 Stochastic framework

We used the above ordinary differential equation framework to develop a stochastic simulation algorithm (SSA or birth–death process) using the direct method described by (Gillespie, 1977), adapted to allow heritable variation in individual traits. Stochasticity occurs in the model as a result of the random selection of birth and death events (demographic stochasticity), and random mutations during reproduction. This framework is apt for testing our assumptions because true extinctions are possible, and evolution occurs as a result of heritable individual variation that emerges from our assumptions about population demography.

We initialize the model with 35 individuals with traits drawn from a uniform distribution with mean 0 and standard deviation 0.3873, which, under a constant environment is a reasonable approximation to the standing variation that our assumptions generate. We determined this by running our simulations in a constant environment and taking the average standard deviation of trait values in the population. Integration of the model starts by first determining the time until the next event, which is randomly sampled from an exponential distribution with mean 1/*E*, where *E* is the sum of the rates of all possible events (birth and death of all *N* individuals in the population):

(7) $$E=\sum_{i}^{N}\left(\left.B\right|\mu_{i}\left.+D\right|\mu_{i}\right)$$

After the current time *t* is updated, the specific event that occurs is determined by randomly choosing among all possible events, weighted according to differences in their rates. For example, the probability that the next event is a death of the *i*th individual is $\left.D\right|\mu_{i}/E$ . If an individual dies, it is removed from the population and the entire process is repeated. If birth of an individual is chosen, the new individual takes the parent's trait with probability 0.9; otherwise, a mutation occurs and the offspring's trait is equal to the parent's trait plus a random value drawn from a uniform distribution with a range of −0.3 to 0.3. This sequence of steps mimics mutation‐limited evolution in an asexual population. A similar eco‐evolutionary framework is described in DeLong and Gibert (2016); however, their approach differs slightly from ours because they first aggregate rates of birth and death to the population level and then randomly assign the individual to experience the event. This results in an underestimate in the response to selection, but leads still to the same equilibrium.

### 2.5 Simulations

We conducted simulations across a log‐linear range of frequencies (*f*) of environmental change. For each frequency of environmental change, we conducted 512 independent replicate simulations. We ran the model for 500 time steps before recording the trait values of each individual, as well as the population size and all simulations continued for a total of 100,000 time steps or until extinction occurred. Trait–environment correlations were computed for the mean trait and environment value using Pearson's correlation coefficients. To provide a basis of comparison, we also conducted simulations where mutation‐driven evolution did not occur. Simulations were conducted using Wolfram *Mathematica* v11.0 on a iMac Pro with 18 Xeon W cores.

Lastly, we conducted simulations utilizing an environment that changes in a sigmoidal manner

(8) $$\mu_{\mathrm{opt}}\left(t\right)=\frac{t-T_{P}}{\sqrt{a+{\left(t-T_{P}\right)}^{2}}}$$

where *a* is 800 and $T_{P}$ or the time at which the environment changes is 600. The slope of the environmental change is determined by *a*, which we chose to be a similar rate of change to that experienced periodically in the sinusoidal environment with *f* = 0.015. We used this additional case to showcase a more traditional type of environmental change to observe evolutionary rescue.

## 3 RESULTS AND DISCUSSION

**Figure 2:**
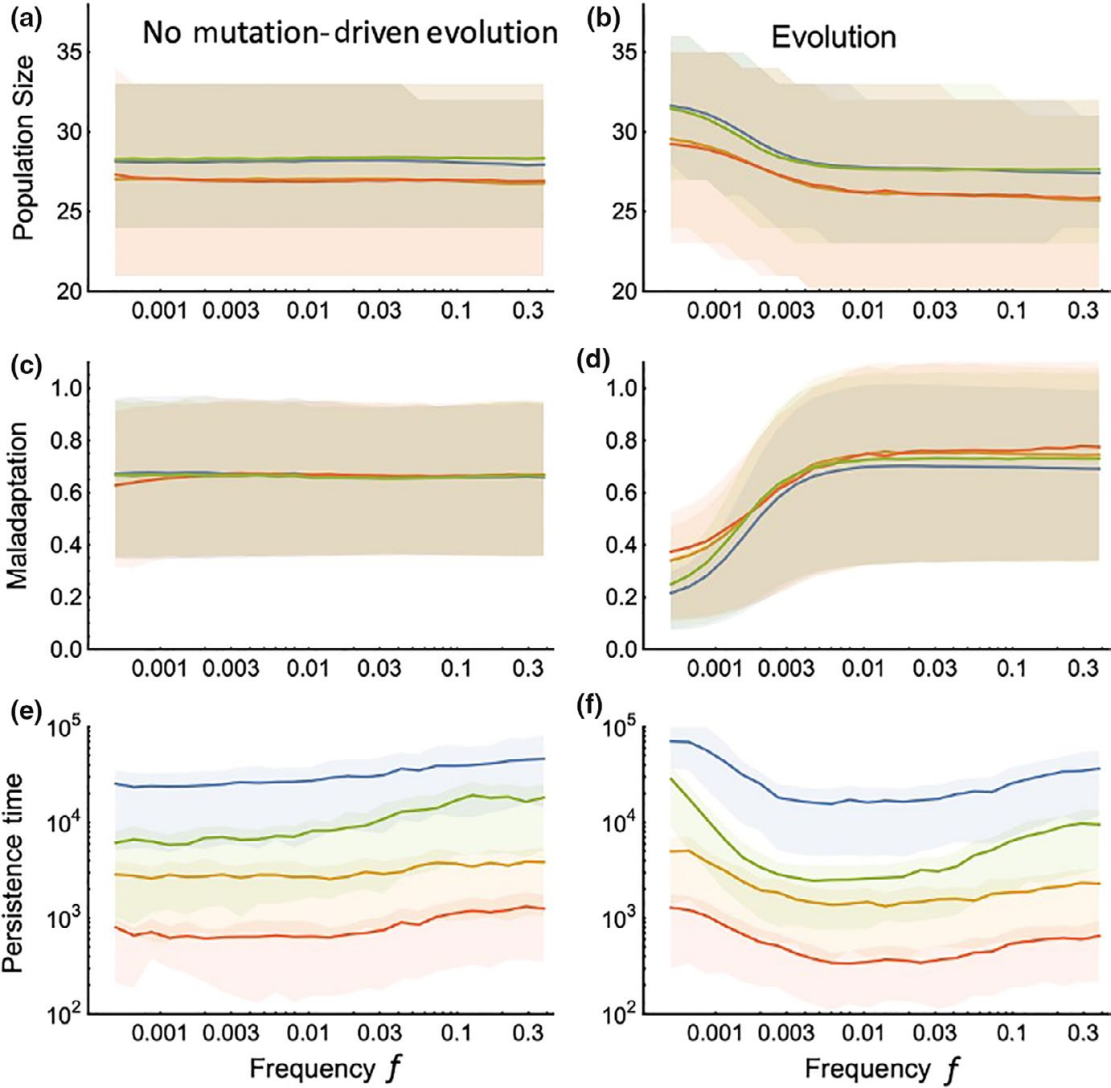
Population dynamics of the four model cases without and with a complete evolutionary dynamic (with and without mutation). For population size (a, b) and maladaptation (c, d), the solid lines give the ensemble means of all model replicates and times and the shaded areas show the 25th and 75th percentiles of the distribution. For persistence time (e, f), the solid lines give the means across model replicates and the shaded areas show the 25th and 75th percentiles of the distribution for persisting populations. Maladaptation is measured as the difference between the mean population trait and the environmental value for persisting populations. The blue line represents Case 1b (environmental change alters the birth rate and population response to density), the green line Case 1a (environmental change alters the birth rate independent of density), the orange line Case 2b (environmental change alters the death rate and population response to density), and the red line Case 2a (environmental change alters the death rate independent of density), as shown in Figure 1

Our results show that evolutionary rescue is affected when the environment influences different demographic rates and processes. We begin by discussing the resulting extinction dynamics when considering populations that cannot undergo evolution, followed by populations that have the capacity for mutation‐driven evolution. The four models we consider here are calibrated to produce the same behavior when the environment is held constant; the population will approach an equilibrium density that is determined by the environment, but is consistent across all cases. At equilibrium, however, the turnover rates (approximated by $\frac{B}{D}$ ) differ among the models in which birth rates vary among individuals and those in which death rates vary (see Figure 1). Consistent differences also emerge among the models incorporating the density‐independent and density‐dependent environmental interaction; particularly at low densities, the effect of trait variation is strongly buffered in the latter cases. These differences give rise to the results depicted in Figure 2.

### 3.1 Demographic results without mutation‐driven evolution

The four models exhibit a consistent ranking of mean persistence time across the entire range of frequencies of environmental change we considered (Figure 2e). Mean persistence was greater in populations whose birth rates (rather than death rates) were environmentally influenced, and when the environment affected the strength of density dependence. In the absence of evolution, the most persistent populations were of the form outlined in Case 1b, followed by Case 1a, where there is a density–environment interaction in the birth rate and where the environment acts on the birth rate independent of density, respectively. These were followed by Case 2b then 2a (the populations where the environment altered the strength of density dependence and acted independent of density on the death rate). This ranking in persistence is easily explained by the ecological differences among the models, considering in particular their behavior when population sizes are small (i.e., as populations are near extinction).

First, populations with birth as the responsive trait persist longer than those with death as the responsive trait due to the greater demographic stochasticity in death models which increases extinction at small population sizes. The intrinsic growth rate of the population is determined by the difference between the birth and death rate, while demographic stochasticity is determined by the sum of the birth and death rate (Nisbet & Gurney, 2003; Palamara, Carrara, Smith, & Petchey, 2016). Although our models are parameterized so that they have the same $K_{A}$ and $K_{B}$ for when B-D=0 , the sum of B and D at these equilibrium points is four times higher in the death models (Case 2a and 2b). Hence, the death models have much higher demographic stochasticity than the birth models (Figure 1), and it is clear that demographic stochasticity increases extinction probability at low population sizes (Lande, 1993; Melbourne & Hastings, 2008). Furthermore, demographic stochasticity increases the variance in population size, as we see in Figure 2 (a,b). High fluctuations in vital rates have been shown to decrease population growth due to an increase in variation in the population growth rate (Jonsson & Wennergren, 2019; May, 1973). Accordingly, species have been shown to be particularly vulnerable to highly variable adult survival, leading to a higher extinction risk (Caswell, Fujiwara, & Brault, 1999; Crone, 2001; Jonsson & Ebenman, 2001; Lande, 1988). Although model results emphasize the importance in specific vital rate change due to selection utilizing matrix modeling approaches (Barfield et al., 2011; Coulson, Kruuk, Tavecchia, Pemberton, & Clutton‐Brock, 2003), they do not analyze the dynamics of small populations with varied distributions of phenotypes as was the goal in the present study.

Second, at low densities, models where the environment interacts with the strength of density dependence maintain higher average (and less variable) population size since maladaptation to the environment has a diminishing impact as population size declines (Figure 1b,d). This is reasonable as populations with highly variable growth rates have been shown to be particularly vulnerable to extinction (Lande & Orzack, 1988; Leigh, 1981). Furthermore, it has been shown with a discrete time model that when the environment is embedded in a density‐dependent term, it produces a multiplicative effect on population size, and these populations have more strongly bounded populations (Ferguson & Ponciano, 2015). As shown in Figure 1b,d, at low population sizes, the density‐dependent environmental effect has lower variation than the density‐independent environmental effect, while the opposite is the case at large population sizes. These differences in variation translate into longer persistence times of the models where environmental change alters the effect of density (Case 1b, 2b) relative to those where environmental change alters the vital rates independent of density (Case 1a, 2a). Although the environmental density effect increases variation at high population sizes, it is favorable when populations are small as they are better able to rebound. Researchers have emphasized the importance of density dependence in population growth of course (Chevin & Lande, 2010; Clutton‐Brock & Coulson, 2002; Holt, 1990), but the effect of whether or not selection alters said density dependence has been emphasized in this study.

**Figure 3:**
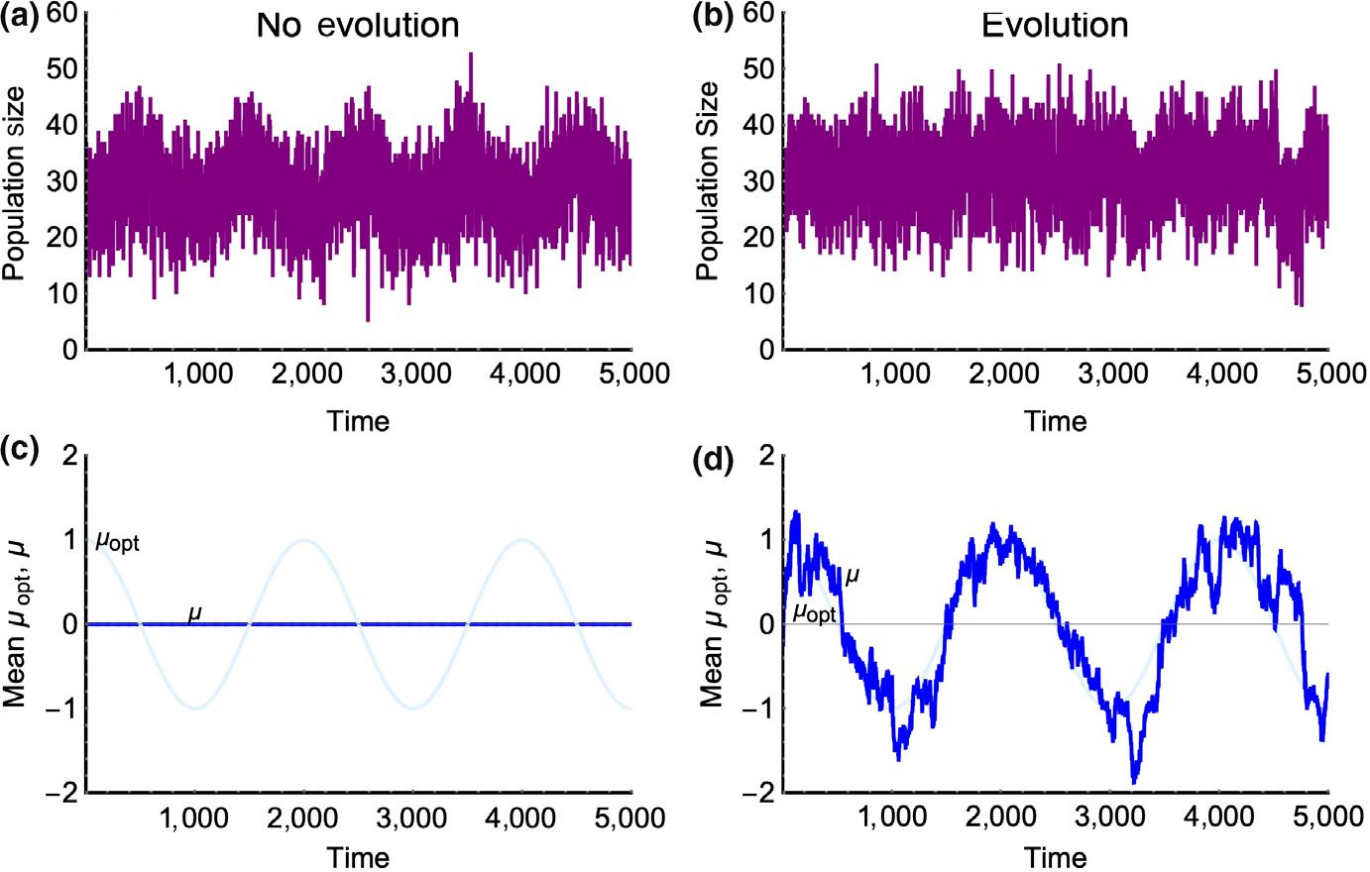
Ecological tracking occurs when the population size (a) exhibits a correlated pattern of variation with the environment (here $\mu_{\mathrm{opt}}$ ) (panels a and c). In this example, all individuals have the same trait value, there is no mutation‐driven evolution, and f=0.0005. Panels b and d show evolutionary tracking where the mean trait in the population closely follows the environment, thereby dampening the ecological response to the environmental variation

All four scenarios exhibit a rising persistence time as the frequency of environmental variation increases. This is driven by a phenomenon known as “ecological tracking”; when a population ecologically tracks its environment, changes in the environment are re‐expressed in the population dynamics as correlated changes in density. Here, where the environment changes sinusoidally, ecological tracking generates population dynamics that exhibit a noisy cycle at the same frequency as the environment (Figure 3a,c); however, the tracking response of population diminishes as *f* increases. In May, 1976; Lande et al., 2003, it is suggested that the system's dominant eigenvalue represents a threshold frequency above which tracking does not occur in the Logistic model, but the exact relationship between tracking and the frequency of oscillations is best described as a continuous sigmoid function (Vasseur, 2007). The stronger tracking response generated at low frequencies of environmental variation leads to greater variation in population density (both above and below the mean) and thus greater extinction risk. This effect has been shown for a variety of ecological scenarios (Heino, Ripa, & Kaitala, 2000; Lande et al., 2003; Schwager, Johst, & Jeltsch, 2006).

### 3.2 Demographic results with evolution

When the full eco‐evolutionary dynamics are present in our models, we find that the persistence ranking of models is maintained; however, all four models demonstrate a *U*‐shaped (rather than monotonic) relationship between the frequency of environmental change and mean persistence times. This *U*‐shaped relationship arises due to the interplay between ecological and evolutionary tracking of the changing environment. Evolutionary tracking occurs when changes in the environment are slow enough that they can be re‐expressed as correlated changes in the mean or modal trait value(s) of the population. Importantly, evolutionary and ecological tracking are interdependent, here forming a link between ecology and evolution. As evolutionary tracking strengthens, ecological tracking is diminished because a population that adapts quickly does not experience the same extent of variation in its vital rates and parameters (See Figure 3b,d). As ecological tracking generally has a negative effect on persistence, evolutionary tracking generates a benefit mitigating the population's response to ecological tracking. Given the assumptions of our model (mutations per birth, mutation effect size, and population size) evolutionary tracking occurs at frequencies of environmental change below approximately f=0.005 . Here, it can be seen that the deviation between traits and the environmental optimum tends to decline at low frequencies (Figure 2d), leading to an increase in the population size and mean persistence times. Variation in population size is not only caused by variation of demographic stochasticity between different vital rates, but also by intraspecific trait variation. Since any individual can give birth in dynamic death models, they have more trait variation in the autocorrelated environments, (low *f*) which increases the effect of maladaptation on their death rate. But as the *f* increases, the effect of maladaptation becomes the same across the models.

**Figure 4:**
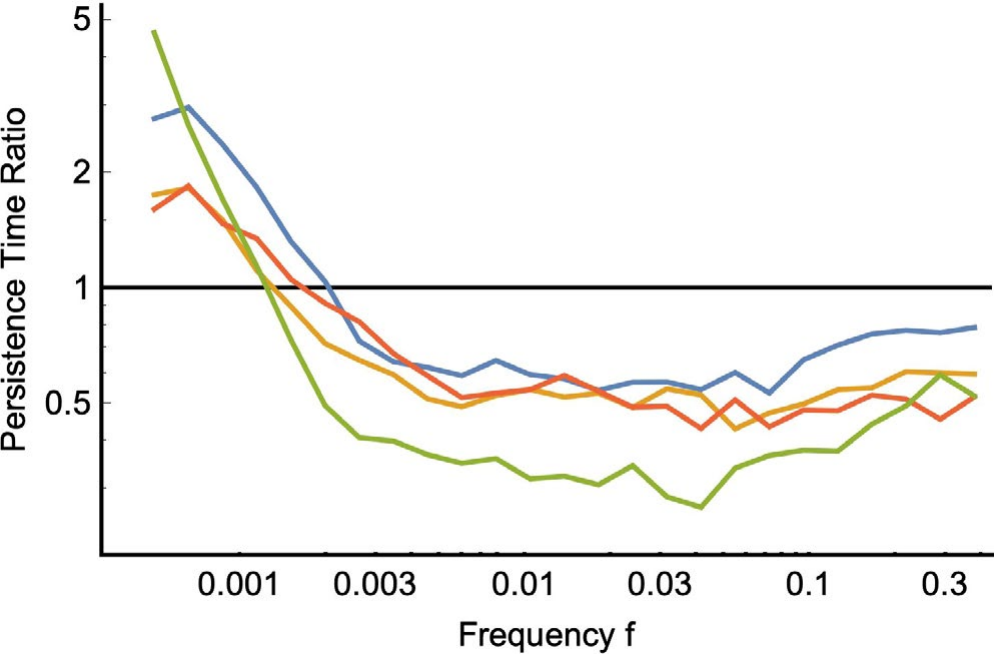
The quotient between the mean persistence time of populations that exhibited evolution and the mean persistence time of populations that did not undergo mutation‐driven evolution. For values above one, evolution was beneficial for persistence, and for those below one, evolution had a negative impact. Evolutionary tracking increased persistence time for populations when the environmental fluctuation frequency was low

The eco‐evolutionary dynamic, that is responsible for an increase in persistence times at low frequencies of environmental fluctuation, also leads to a reduction in persistence time at intermediate and high frequencies (Figure 4). This reduction is due to mutational loading (Higgins & Lynch, 2001) which is here exacerbated by the fact that mutations which might be immediately favorable in the population become quickly deleterious as the environment oscillates. This confounding kind of evolution is most likely to occur at intermediate frequencies, where complete evolutionary tracking is unlikely, but random chance allows momentary “misleading” evolutionary changes to occur. Consistent with this idea, we see a slight inflation of the mean and range of maladaptation in our eco‐evolutionary models (Figure 2c) relative to those without mutation‐driven evolution. All of our models transition from a detrimental, to a beneficial effect of the eco‐evolutionary dynamic near. Determining how this threshold relates to the life‐history parameters of natural populations will provide important information about the potential for evolution to buffer populations from extinction in oscillating environments. Note that in Figure 2c, the mean line is slightly decreased at low f for the death models. This is due to the higher trait variation exhibited in these models as previously discussed, causing a larger deviation from the optimal trait condition.

### 3.3 Consequences of environmental effects on different demographic rates

In natural populations, we see that the demographic rates that are selected upon, and how density dependence responds varies. Some populations may respond to environmental change in a density‐independent way as in Cases 1a, 2a (Brewer & Peltzer, 2009; Dempster, 1983) while some are likely to show an increase in the intensity of density dependence as in Cases 1b, 2b (Aanes et al., 2000; Coulson et al., 2001), with varied key demographic rates (birth or death). These results emphasize the importance of taking specific demographic parameters into account into our models in the light of evolutionary rescue. Furthermore, these results suggest that environmental change that primarily causes an increase in mortality independent of density will be the most destructive to natural populations (Case 2a). We see dynamics such as this when environmental changes drive populations to physiological limits, natural disasters, severe weather, and pollution. For example, a change in oxygen composition in a marine ecosystem may affect a population regardless of density (Brewer & Peltzer, 2009), or an increase in heavy metal contamination may similarly increase mortality regardless of population size (Santala & Ryser, 2009).

According to our results, the populations that will benefit the most from evolutionary rescue will be those whose fecundity responds to an environmental change in a density‐dependent way. This may be exemplified in cases where the availability of, or access to, resources is altered by environmental change. This leads to an interaction between the deleterious effect of a mismatched environment and competition; as population size decreases and competition for resources is relaxed, the effect on demographic rates weakens. This is similar to the environment by competition covariance that is essential to maintaining positive invasion growth rates in the storage effect (Chesson 2000). Note that density dependence can also decrease due to environmental change in areas where the change is favorable (take the case of invasive species and pests), further increasing persistence potential (Ouyang et al., 2014). From these results, we recommend that long‐term studies incorporate fine demographic data when feasible. Further analysis should be done to fine tune the relevant parameters that play a role in evolutionary rescue, so that we may one day be able to predict and promote evolutionary rescue in the wild.

### 3.4 Consequences of our model assumptions

Our modeling framework assumes asexual reproduction and a link between the environment and demographic parameter that is controlled by a single trait. Most empirical and theoretical work suggests that sexual recombination can lead to an increased rate of evolution, as it is beneficial when mutations are common and have a small effect size (Crow & Kimura, 1965). Recombination can also pose the opposite effect by breaking up favorable gene combinations, or allowing maladaptive traits to persist longer in the population, leading to a greater genetic load on population fitness (Uecker & Hermisson, 2016). Thus recent studies show a nonlinear effect of recombination on evolutionary rescue (Uecker, 2017; Uecker & Hermisson, 2016). Incorporating recombination to assess any differences in outcome will surely be relevant given the diversity of mating systems in nature. Furthermore, singular step mutations are what allow the population as a whole to track the changing environment, as opposed to a genotype phenotype mapping that is not one to one. This may be representative of populations with a narrow genetic basis for which adaptation to the environment can occur, such as what has commonly been seen in drug resistance (MacLean, Hall, Perron, & Buckling, 2010). That being said, in nature, some cases of environmental change will surely require multiple traits to evolve for the population to persist. The utility of this model though is that it is comparative, it is likely we will see the same trends in a multi‐trait model but this will surely be fruitful to investigate as we bring our models towards realism. This will become even more relevant with the incorporation of species interactions. Competition can both inhibit and promote evolutionary rescue in different cases (Osmond & de Mazancourt, 2013) and has shown to be a relevant component in the study of population persistence.

**Figure 5:**
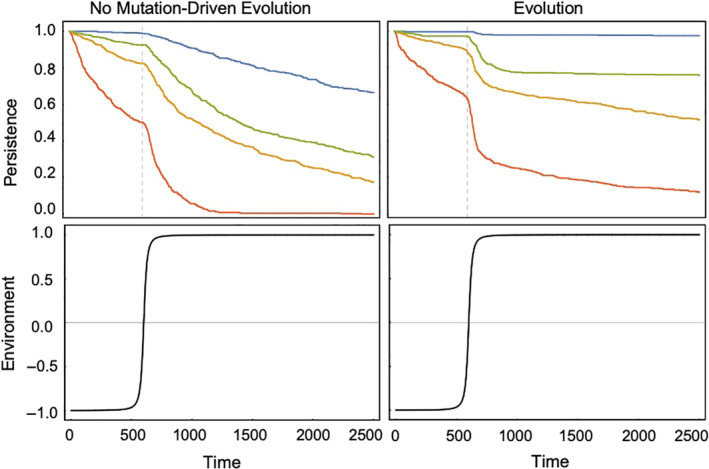
The proportion of persisting populations over time. These plots portray a typical evolutionary rescue scenario with a sigmoidal environment as opposed to the fluctuating environment shown in the previous figures. The top panels depict the proportion of surviving populations over time out of 512 replicates for Case 1a (green), 1b (blue), 2a (red), and 2b (yellow)

Lastly, the environment in this model lacks environmental stochasticity, which has been shown to play a role in the potential for populations to evolve to track the changing environment (Fey & Wieczynski, 2017; Lande et al., 2003; Ovaskainen & Meerson, 2010). But, because we first utilize a fluctuating environment instead of the single step change commonly utilized in evolutionary rescue studies, we are able to characterize the ability for a population to continuously adapt to a changing environment. In this way, we are able to see populations undergoing evolutionary rescue again and again, in order to better understand the mechanisms underlying this dynamic. In environments undergoing noncyclic changes, the rate and extent of environmental change together form a critical axis on which the success of evolutionary rescue (or more appropriately eco‐evolutionary rescue) can be measured. Generally, the potential for eco‐evolutionary rescue is assessed using a singular environmental change, for example, from low to high concentrations of salt, or cold to warm temperatures, (Crump et al., 2004; Doebeli & Dieckmann, 2003; McCain & Grytnes, 2010) and the typical pattern of population and trait dynamics are easily explained using the concepts of ecological and evolutionary tracking applied above; when traits are able to track the environmental change quickly enough, ecological changes are dampened enough to prevent extinction. Thus, our model, which incorporates a cyclic environmental change, is a useful predictor of how different assumptions about life history will alter the propensity of eco‐evolutionary rescue. We confirm that our results are not an outcome of this cyclic environment, as the same persistence ranking results from a sinusoidal shift in the environment (Figure 5).

The study of evolutionary rescue has increased notably in the past decade, and although we have elucidated a reduced set of relevant factors, the interplay between demography and evolutionary rescue is still largely unknown. We show that models with varied dynamic demographic parameters with the same carrying capacities and initial conditions have different probabilities of undergoing evolutionary rescue following environmental change. Therefore, comparative evolutionary demography provides a lens with which we can understand how different populations may be more or less likely to persist alongside environmental change. As emphasized in previous studies, evolutionary rescue in these models occurs when the rate of environmental change, or the fluctuation frequency, is slow enough for the population to evolutionarily track the changing trait optimum as shown in Figure 3b,d (Lindsey et al., 2013; Perron, Gonzalez, & Buckling, 2008). Although the current model does not take into account spatially heterogeneous environments or interspecific competition, it provides a starting point to better understand the interplay between evolutionary demography and evolution to a changing environment. We find that changing the demographic parameter that selection acts on, as well as the way in which selection alters density dependence, changes a populations propensity to avoid extinction via evolutionary rescue.

## 4 CONCLUSION

In order to minimize extinction of natural populations alongside changing environmental conditions such as climate change, we must be able to make decisions without complete data describing future phenomena. It is therefore vital to create theory that can aid scientists and wildlife managers alike in understanding how natural populations respond to escalating rates of environmental challenge. This includes techniques utilizing the population data we already have, to use the past as a proxy for the future, as well as techniques utilizing our understanding of evolution to form ideas of how populations can adapt and how we can help them to adapt to persist into the future.

We show that when evolution is occurring in a system, the extinction probabilities vary given different dynamic demographic parameters. This comes into play in how well a population can evolve to have high fitness in a changing environment and the ability of a population to rebound from small population sizes. Our findings show the importance of explicitly incorporating environmental change and density dependence into equations describing population demographic rates. In our study, the environment provides the selective pressure on individuals, and unlike in previous work, the shape of this selective pressure is shown to differ between commonly used models. This result would not have been shown had we focused on a purely ecological or evolutionary model, this interplay is what allows us to make novel insights into if and how population persistence will be altered by climate change. Furthermore, incorporating selection and trait evolution into models on ecological time scales is an important research priority. This work shows that natural populations that have different key demographic rates will likely respond differently to climate change, and this information should be explicitly incorporated into models that predict extinction due to climate change.

## CONFLICT OF INTEREST

None declared.

## AUTHOR CONTRIBUTION

**Anna Christina Vinton:** Conceptualization (lead); Data curation (lead); Formal analysis (lead); Methodology (lead); Project administration (lead); Visualization (lead); Writing‐original draft (lead); Writing‐review & editing (lead). **David Alan Vasseur:** Conceptualization (supporting); Data curation (supporting); Formal analysis (supporting); Investigation (supporting); Methodology (supporting); Project administration (supporting); Supervision (equal); Validation (supporting); Visualization (supporting); Writing‐original draft (supporting); Writing‐review & editing (supporting).

## Data Availability

A copy of our computer code is on DRYAD: https://doi.org/10.5061/dryad.w6m905qmg.
